# Effects of aqueous extract from Asparagus officinalis L. roots on hypothalamic-pituitary-gonadal axis hormone levels and the number of ovarian follicles in adult rats

**Published:** 2016-02

**Authors:** Hojatollah Karimi Jashni, Hossein Kargar Jahromi, Ali Ghorbani Ranjbary, Zahra Kargar Jahromi, Zahra Khabbaz Kherameh

**Affiliations:** 1 *Zoonoses Research Center, Jahrom University of Medical Sciences, Jahrom, Iran.*; 2 *Young Researchers and Elite Club, Kazerun Branch, Islamic Azad University, Kazerun, Iran.*

**Keywords:** *Asparagus officinalis*, *Hormones*, *Ovary*, *Oogenesis*, *Rat*

## Abstract

**Background:**

Asparagus is a plant with high nutritional, pharmaceutical, and industrial values.

**Objective:**

The present study aimed to evaluate the effect of aqueous extract of asparagus roots on the hypothalamic-pituitary-gonadal axis hormones and oogenesis in female rats.

**Materials and Methods:**

In this experimental study, 40 adult female Wistar rats were divided into five groups, which consist 8 rats. Groups included control, sham and three experimental groups receiving different doses (100, 200, 400 mg/kg/bw) of aqueous extract of asparagus roots. All dosages were administered orally for 28 days. Blood samples were taken from rats to evaluate serum levels of Gonadotropin releasing hormone (GnRH), follicular stimulating hormone (FSH), Luteinal hormone (LH), estrogen, and progesterone hormones. The ovaries were removed, weighted, sectioned, and studied by light microscope.

**Results:**

Dose-dependent aqueous extract of asparagus roots significantly increased serum levels of GnRH, FSH, LH, estrogen, and progestin hormones compared to control and sham groups. Increase in number of ovarian follicles and corpus luteum in groups treated with asparagus root extract was also observed (p<0.05).

**Conclusion:**

Asparagus roots extract stimulates secretion of hypothalamic- pituitary- gonadal axis hormones. This also positively affects oogenesis in female rats.

## Introduction

Estrogen and progesterone are female sex hormones, which regulate reproductive, skeletal, cardiovascular, and central nervous systems. Estrogen is secreted in normal ovarian cycle in follicular phase from granulosa cells. Progesterone is secreted from corpus luteum during ovulation. Luteinal hormone (LH) and follicular stimulating hormone (FSH) are glycoprotein hormones released from anterior pituitary gland. Gonadotropin releasing hormone (GnRH) is a peptide secreted from hypothalamic neurons which is secreted in pulsatile pattern and releases FSH and LH from the pituitary gland (1, 2).

Herbs positively increase fertility and resolve such issues as hormonal imbalances, impotence, etc. Asparagus officinalis L. is an important garden vegetable plant, which is cultivated in temperate and subtropical areas. Asparagus is an herbaceous, perennial, and diclinous plant belonging to asparagus order, asparagaceae family and asparagus genus (3). This species is an economically important genus of asparagus with high nutritional, pharmaceutical and industrial values. Asparagus cultivation is growing due to rich anti-cancer compounds and antioxidants, such as saponin, aspartic acid, rotin, protodioscin, glutathione, flavonoid, and A, B, C, and E vitamins as well as zinc and fiber content (3-5). The extract of this plant contains amino acids and minerals that can overcome lethargy and malaise and protect liver cells against toxins (6). In addition, asparagus is rich in vitamin K and folate (vitamin B9). Folate in asparagus reduces congenital malformations during pregnancy. Asparagus boosts sexual stamina (7). Asparagus also contains phyto-estrogens, which are bioactive drugs (8-10). These compounds are also known as A-I asparagoides. Despite medicinal application of asparagus for menstrual disorders in women, there is no evidence sufficient to support this claim. Steroid saponin is phytoestrogen compounds extracted from asparagus roots (11). 

The results of several studies showed that saponins reduce damaging effects of chemotherapy on sexual organs. Saponins also possess antioxidant properties (12). Thus, according to presence of multiple compounds affecting gonadotropic and ovarian hormones in the asparagus extract such as steroidsaponins, vitamins, and amino acids, the present study aimed to evaluate the effect of aqueous extract of asparagus roots on the hypothalamic- pituitary- gonadalaxis hormones and oogenesis in Wistar rats.

## Materials and methods

This experimental study was conducted at Jahrom Medical University during 2015. 40 adult female Wistar rats with an average weight from 180-200 gr were used. The rats were kept in room for animal nurturing in Shiraz Medical Sciences University for a week to adapt to environment. Ethical principles and considerations in terms of laboratory animals were approved by the University and were observed by the authors in the present study.

Throughout the study, animals were kept in 12 hr dark/light and an ambient temperature from 22-25^o^C. The animals had free access to food and water. The animals were randomly divided to 5 groups consisting control, sham and three experimental groups (n=8/each). Experimental groups received different doses of asparagus extracts.

Fresh roots of Asparagus officinalis were collected from the Medicinal Plants Garden belonging to Shiraz University (Shiraz, Iran). To prepare asparagus extract, the roots were completely cleaned and dried in the laboratory as powder form. The resultant powder was mixed with 96% ethyl alcohol at a ratio of 5 times the size of the plant. The mixture was stirred in rotodoxy device for 24 hr at room temperature to obtain a uniform solution. The solution was filtered and dried for 48 hr at ambient conditions to obtain a solid extract with no alcohol. Then, 100, 200 and 400 mg of the solid extract were dissolved in 1 ml of distilled water twice. The solutions were kept at refrigerator until being used (13).

The control group did not receive any dosage of asparagus extract. The sham group was orally prescribed 1 ml of distilled water with respect to body weight. Experimental groups orally received minimum (100 mg/kg), moderate (200 mg /kg), and maximum dose (400 mg /kg) of asparagus roots aqueous extract in daily manner for 4 weeks with respect to body weight, respectively. At the end animals were weighted and 5 cc blood was directly taken from the heart of animals using a syringe (under anesthesia by diethyl ether). The serums were centrifuged (at 3000 rpm for 15 min) and were stored in the freezer at -20^o^C. 

ELISA kits specific for rats were used (Manufactured by Biovendor Corporation in Czech Republic) for measuring GnRH (Cat n. E0185RA), FSH (Cat n. E0182RA), LH(Cat n. E0179RA), estrogen (Cat n. DKO003), and progesterone (Cat n. DKO0015) hormones. 

**Ovarian morphology**

Abdominal incision was made in rats. The ovaries were isolated with scalpel and forceps and cleaned fat. The ovaries were weighted with a digital scale and were washed with saline. Then, each ovary was kept for 14 days in a tube containing 3% formaldehyde. Ovaries were imbedded in paraffin, cut in 5-μm sections, and stained with hematoxylin and eosin. 

**Ovarian slices**

For this purpose, 10% of the ovary was non-random sliced (14). In summary, in the first section that follicles were observed, follicle count was done. Then after every ten sections it was selected and re-counting was done. It continued to count the last last slice of ovary and then obtained data were collected. In this way, 10% of total sections were counted non- random (15). 

The number of primordial, primary, secondary, graph, atretic follicles and corpus luteum were counted by Nikon optical microscope with 40 and 400 magnifications among the prepared slides. The average number of follicles in each group was specified and compared with other groups.

**Statistical analysis**

All data are shown as Mean±SE. The ANOVA test was used for all groups. Duncan's test was used to understand the difference between means in cases where the difference between groups was significant. Statistical analysis was performed using SPSS version 21. The significance level was considered as p<0.05.

## Results

The results showed GnRH hormone levels in experimental groups has been significantly increased compared with control groups (p≤0.001). In moderate and high dose groups, FSH levels were significantly higher than control and sham (p≤0.001, p≤0.001, respectively), while LH levels were increased in high dose group (p≤0.001) (Table I). Also, in experimental groups, estrogen and progesterone serum levels were significantly increased (p<0.05) (Table I).

**Morphology**

**Figure 1:**
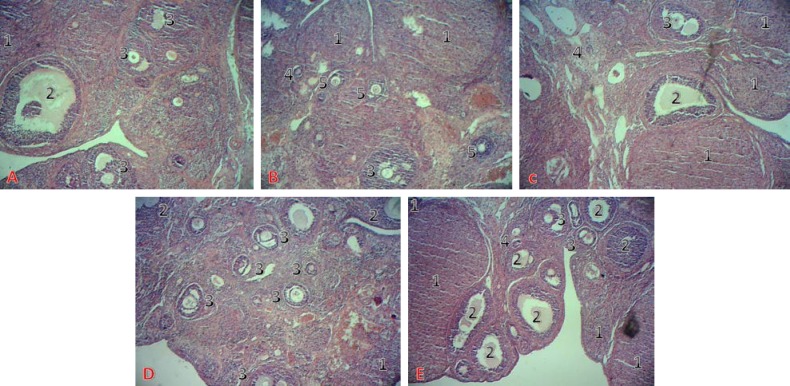
A. Normal histological structure of ovary with different follicles in the cortex in the control group, B. Ovary tissue in sham group, C. Ovary tissue of rats with no significant change in the 100 mg/kg Asparagus officinalis extract group compared to control group, D. Numerous primordial, primary, graph follicles in the ovarian cortex of rats in the 200 mg/kg Asparagus officinalis extract group, E . Numerous primordial, primary, graph follicles and Corpus luteum in the ovarian cortex of rats in the 400 mg/kg Asparagus officinalis extract group (Hematoxylin-Eosin ×40

The results of counting the number of ovarian follicles in different groups are shown in table II. Based on results, number of primordial, primary, and graph follicles in moderate and high dose groups significantly increased (p≤0.001). No significant difference was observed between mean numbers of atretic follicles in experimental groups compared to control and sham. The results also showed that mean number of corpus luteum in high dose experimental group significantly increased compared to control group (p<0.05) (Table II). Hyperemia and ovarian tissue destruction and fibrosis were not observed (Figure 1c, 1D, 1E).

**Table I:** GnRH, FSH, LH, estrogen, and progesterone levels in experimental and control groups

**variable**	**Control group**	**Sham group**	**Experimental group **			**p-value** *
			**100 mg/kg**	**200 mg/kg**	**400 mg/kg**	
GnRH (IU/L)	80.67 ± 8.7 ^a^	85.02 ± 8.5 ^a^	112.6 ± 3.5 ^b^	114.7 ± 12.9^b c^	120.5 ± 12.08 ^b^	<0.001
FSH (IU/L)	0.28 ± 0.21 ^ab^	0.27 ± 0.23^a^	0.32 ± 0.46 ^b^	0.37 ± 0.46^ c^	0.4 ± 0.75 ^c^	<0.001
LH (IU/L)	125.1 ± 4.1 ^ab^	120.2 ± 5.7^a^	131.2 ± 5.6 ^bc^	13 1.1 ± 4.1 ^bc^	136.1 ± 5.7 ^c^	<0.001
Estrogen (Pg/ml)	13.9 ± 2.05 ^a^	13.4 ± 2.2^a^	35.7 ± 4.01 ^b^	62.9 ± 10.5 ^c^	70.3 ± 8.4 ^d^	<0.001
Progesterone (ng/ml)	17.6 ± 3.2 ^a^	16.03 ± 2.3^a^	34.6 ± 5.7 ^b^	35.5 ± 2.9 ^b^	40.91 ± 4.6 ^c^	<0.001

- All data were presented as Mean±Standard Error.

GnRH: Gonadotropin releasing hormone

FSH: follicular stimulating hormone

LH: Luteinal hormone

* Duncan test, the means in the rows with at least one common letter were not significantly different at 5% level.

**Table II:** Comparison of the number of ovarian follicles in the experimental groups with the control group

**Variables**	**Control group**	**Sham group**	**Experimental group**			**p-value** *
			**100 mg/kg**	**200 mg/kg**	**400 mg/kg**	
Primordial follicles	5.3±1.06 ^a^	5±0.92 ^a^	4.2±0.70 ^a^	7.8±1.9 ^b^	8.1±3.1 ^b^	0
Single-layer primary follicle	3.7±0.70 ^a^	3.8±0.64 ^a^	4±1.3 ^a^	5.8±1.1 ^b^	6.6±2.1 ^b^	0
Multilayered primary follicle	2.7±0.70 ^ab^	2.6±0.74 ^a^	3±0.53 ^ab^	3.3±0.51 ^b^	4.2±0.70 ^c^	0
Secondary follicle	3.6±0.91 ^ab^	3.1±0.99 ^a^	3.6±1.06 ^ab^	4.2±1.03 ^b^	4.2±0.70 ^b^	0.111
Graph follicle	2.5±0.53 ^a^	2.2±0.46 ^a^	2.1±0.35 ^a^	3.5±0.53 ^b^	4.5±053 ^c^	0
Corpus luteum	3.7±1.1 ^ab^	3.3±0.46 ^a^	4.1±0.83 ^ab^	4.3±0.74 ^b^	5.7±1.03 ^c^	0
Atretic follicle	0±0 ^a^	0.2±0.46 ^a^	0.1±0.35 ^a^	0.2±0.46 ^a^	0.3±0.51 ^a^	0.426

- All data were presented as Mean±SE.

* - Duncan test, the means in the rows with at least one common letter were not significantly different at 5% level.

## Discussion

Based on the results, dose-dependent aqueous extract of asparagus roots increases levels of hypothalamic- pituitary- gonadal axis hormones in female rats. The maximum increase in GnRH, FSH, LH, estrogen, and progesterone hormonal levels was observed in experimental group receiving high dose (400 mg/kg) of the extract.

Asparagus roots are rich in such amino acid compounds and derivatives as aspartic acid and arginine (18). Aspartic acid stimulates secretion of gonadotropin- releasing and luteinizing hormones. Tests showed that this amino acid regulates synthesis of luteinizing hormone by looped guanosine mono phosphate through as second messenger in pituitary (19). Arginine in asparagus is also converted to nitric oxide, which is one of the most important factors controlling the release of LH and FSH (18). Nitric oxide producing neurons directly secrete GnRH and consequently LH and FSH (18-20). 

It seems that presence of phytoestrogen compounds in asparagus extract is also effective in increasing levels of ovarian hormones. Phytoestrogens are natural plant-derived compounds whose functions are similar to estrogen. Steroid saponins such as sarsaponin, protodioscin, and diosgenin are the most likely estrogenic components extracted from asparagus roots (21). These compounds also act as a precursor of progesterone and increase secretion of this hormone (22, 23).

Asparagus roots are rich in such minerals as calcium, magnesium, phosphorus and zinc (4, 5). The presence of minerals in follicular fluid regulate growth of follicles and steroidogenesis. Minerals not only act a cofactor in different enzymatic activation systems for growth and maturation of oocytes, but also affect ovarian function and fertility (24, 25). In this study, histological analysis also suggested increasing number of ovarian follicles and corpus luteum as well as insignificant increase in the number of atretic follicles in the experimental groups treated with asparagus extracts. This result is consistent with an increase in hypothalamic-pituitary-gonadal axis hormones. Growth and development of ovarian follicles by proliferation and differentiation of granulosa cells are affected by FSH. 

In early stages of follicular development (initial and primary), granulosa cells are proliferated slowly but granulosa cells of preantral follicles respond to stimulation of FSH and secrete large amounts of estradiol (26). Thus, an increase in estrogen due to an increase in number of follicles was not unexpected in the present study. Maximum increase in graph follicles was observed in the experimental group receiving 400 mg/kg dose of the extract. Maximum estrogen level was also observed in this group. 

Increased number of corpus luteum also was corresponded with an increase in LH. LH affects theca corpus luteum cells, which consequently increases synthesis of progesterone hormone (26). Therefore, an increase in progesterone level was not unexpected according to increased number of corpus luteum in study.

## Conclusion

According to the results, it seems that asparagus root extract directly stimulates gonadotropin hormones and improves the process of oogenesis. Also it consequently increases the number of follicles and ovarian hormones.
